# Supplementary material for: Health Care Needs in School-Age Refugee Children
Source: Int J Environ Res Public Health. 2019 Nov 1;16(21):4255. doi: 10.3390/ijerph16214255 (PMC6862330; doi:10.3390/ijerph16214255)
Supplement: Supplementary file 1 [file ijerph-16-04255-s001.pdf]

Appendix.

Malmö city

MEDICAL ASSESSMENT OF NEWLY ARRIVED CHILDREN FROM ABROAD

Date:

Name	0		
Surname	0		
ID	0		
Address:	0		
Postal code	0	Phone	
Country of birth	0	Mother tounge	
Date of arrival in Sweden			
Place of arrival in Sweden			

If yes on any of these question-immediate referral to Refugee health clinic

Affected general condition	Yes	No
Jaundice(eyes and/or skin)	Yes	No
Diarrhea	Yes	No
Longstanding cough	Yes	No
Longstanding fever	Yes	No
Acute contagious skin disorder (scabies etc)	Yes	No
Mouth and throat infection	Yes	No
Abnormal fatigue	Yes	No

Anemia, marked pallor	Yes	No
Previous hospital admission with blood transfusion	Yes	No
Night sweat	Yes	No
Family relation has ongoing infection	Yes	No

If yes on any of these questions, communicate result to school nurse/physician in the school the student will attend

History of poor growth	Yes	No
History of poor eyesight	Yes	No
History of impaired hearing	Yes	No
History or sign of disability	Yes	No
History of severe psychological trauma	Yes	No
History of other psychological problem	Yes	No
Caretaker has concern about the child's health	Yes	No

Information to school health nurse at the receiving school

Does the caretaker think the child is in good health?<br>Comment	Yes	No
Any ongoing medication?<br>Comment	Yes	No
History of allergies?<br>Comment	Yes	No

Weight (kg):								
Height (cm):								
Back:	Grade:							
Vision:	Right:		Left:					

Hearing:	Right:		Left:					
Teeth:								
Sleep:								
Food:								
BCG-SCAR	Yes. No.							
Testicles:	Yes, according to caretaker No, according to caretaker. Unknown							
Age of menarche:		Year:						
Previous vaccinations:								
Other:								

Referrals

Student referred to:<br>Comment
Communication with attending school nurse/physician<br>Comment
Screening made by:

Student can attend classes

Date:	
Signature:	
